# Can we use gonadotropin plasma concentration as surrogate marker for BMI-related incomplete estrogen suppression in breast cancer patients receiving anastrozole?

**DOI:** 10.1186/s12885-017-3208-6

**Published:** 2017-03-28

**Authors:** A. Oberguggenberger, V. Meraner, M. Sztankay, B. Beer, G. Weigel, H. Oberacher, G. Kemmler, T. Czech, B. Holzner, L. Wildt, B. Sperner-Unterweger, M. Daniaux, M. Hubalek

**Affiliations:** 1 Department of Psychiatry, Psychotherapy and Psychosomatics, Medical University of Innsbruck, Anichstraße 35, 6020 Innsbruck, Austria; 2 Central Institute for Medical and Chemical Laboratory Diagnostics, Innsbruck Medical University, Anichstrasse 35, 6020 Innsbruck, Austria; 3 Institute of Legal Medicine and Core Facility Metabolomics, Innsbruck Medical University, Muellerstrasse 44, 6020 Innsbruck, Austria; 4 Department of Obstetrics and Gynecology, Innsbruck Medical University, Anichstraße 35, 6020 Innsbruck, Austria; 5 Department of Gynecological Endocrinology and Reproductive Medicine, Innsbruck Medical University, Anichstrasse 35, 6020 Innsbruck, Austria; 6 Department of Radiology, Innsbruck Medical University, Anichstrasse 35, 6020 Innsbruck, Austria

**Keywords:** Breast cancer, Gonadotropins, Body mass index, Estradiol, Aromatase inhibitor

## Abstract

**Background:**

BMI has been suggested to impact on estrogenic activity in patients receiving anastrozole resulting in a reduced treatment efficacy in obese women. Current evidence in this regard is controversially discussed. Since estradiol is inversely correlated with gonadotropins it can be assumed that an impact of BMI is also reflected by gonadotropin plasma concentrations. We aim at investigating the impact of BMI on the hormonal state of breast cancer (BC) patients receiving anastrozole indicated by LH, FSH and SHBG as well as estradiol.

**Methods:**

We determined gonadotropin-, estradiol- and anastrozole- serum concentrations from postmenopausal, early stage breast cancer patients receiving upfront anastrozole within routine after care. Gonadotropin plasma concentrations were derived from the routine laboratory examination report. A liquid chromatography tandem mass spectrometry method was used for the measurement of anastrozole serum concentrations. BMI was assessed within the routine after-care check-up.

**Results:**

The overall sample comprised 135 BC patients with a mean age of 65.3 years. BMI was significantly correlated with LH, FSH and SHBG. This association was neither influenced by age nor by anastrozole serum concentrations according to the regression model. Despite aromatase inhibition 12% of patients had detectable estrogen levels in routine quantification.

**Conclusion:**

Obese women have an altered hormonal situation compared to normally weight women under the same dose of anastrozole. Our study findings are a further indicator for the relevance of BMI in regard of anastrozole metabolism and possible estrogenic activity indicated by gonadotropin plasma level.

## Background

The observation of interindividual differences regarding aromatase inhibitor (AI) metabolization has moved research to the question of who benefits most from this adjuvant treatment for breast cancer (BC) [1]. The search for differential factors influencing treatment efficacy [2] has recently identified the issue of BMI as an important factor related to AI metabolization. In the ABCSG-12 study an increased BMI has been associated with an increased risk for disease recurrence and mortality in premenopausal women receiving goserelin and anastrozole [3]. However, this effect was not observed in the tamoxifen treated group. These results have been supported by others [4, 5]. However, also the ABCSG-6a trial, investigating extended aromatase inhibitor treatment (AT), proved BMI to predict outcome benefit in favour of normally weight women. The relevance of BMI regarding AT has also been illustrated by the authors by investigating the pharmacokinetic aspects of anastrozole in relation to BMI indicating an obesity related anastrozole metabolization [6]. Based on the well-known association of obesity and increased overall estrogenic activity resulting in higher baseline estrogen values compared to normal weight women [7], it has been suggested that plasma estrogen levels might be insufficiently suppressed in obese women when following standard AI dosing recommendations. This hypothesis was supported only recently by Folkerd and colleagues [8] who demonstrated that BMI is related to levels of estrogen suppression. Obese women had not only greater levels of estrogen at baseline but also within the course of treatment. However, this was significant only for the letrozole group. Additionally, information on AI serum concentrations has been lacking which might be a mediating factor in this regard. Finally, the measurement of estradiol has been challenging due to a lack of assays with adequate functional sensitivity [9] which is another limiting factor in this study.

Based on the above-mentioned observations and the well-known inverse association of estradiol and gonadotropins [10–12], it can be assumed that the potential influence of BMI is also reflected by levels of gonadotropin plasma concentration. Gonadotropins might act as a surrogate marker for estrogenic activity that could be easily determined in routine laboratory analysis.

We, thus, aim in this study to investigate the impact of BMI on the hormonal state in a group of postmenopausal BC patients receiving endocrine treatment with anastrozole in routine aftercare. This includes the determination of gonadotropin (LH, FSH, Sex hormone binding globulin-SHBG) and estradiol plasma levels considering anastrozole serum concentrations. This analysis supplements the work of the authors previously published [6].

## Methods

The Ethics Committee of Medical University of Innsbruck approved the study: Study number UN3648, 277/4.9.

### Sample

For the study presented herein, data were derived from a sample of BC patients treated at the Outpatient Unit of the Department of Gynaecology and Obstetrics Medical University Innsbruck, which has been published previously [6, 13]. From this overall sample of 242 BC patients receiving anastrozole, only postmenopausal patients were included in the pharmacokinetic or endocrine analysis to provide group homogeneity (*n* = 7 were excluded from the original sample as done in the analysis previously reported [6]). From the remaining 235 postmenopausal patients information on the hormonal status was available for 135 patients. Consequently, a final sample of 135 postmenopausal BC patients receiving anastrozole was included in the analysis. No group differences between patients with vs. without hormonal data were found regarding clinical or sociodemographic variables. Logistic reasons account for the missing values.

As described previously [6, 13] eligible patients were identified by searching the medical records and included in the study at one of their routine follow-up visits. All participating patients provided written informed consent. After approval of study participation additional blood samples for the determination of anastrozole plasma concentration as well as the hormonal status (estradiol, gonadotropins) were collected in the course of the patients’ routine blood examination. BMI was assessed within the routine after-care check-up. Patient characteristics such as age, menopausal status and clinical variables were derived from the medical records. Sociodemographic and clinical variables are routinely up-dated at every patient visit. The patient’s menopausal status is routinely determined at the clinical appointment by use of laboratory analysis of hormones as well as clinical exploration (clinical indicators for menopause) and is recorded in the medical history.

### Analytical procedure

#### Analysis of anastrozole levels

A liquid chromatography-tandem mass spectrometry method was used for the determination of anastrozole serum concentrations. Please find details on the analytical procedure for the determination of anastrozole serum concentrations elsewhere (Beer et al. 2012). Human plasma samples were processed with a solid-phase extraction (SPE) procedure on polymeric mixed-mode columns (Strata X-C, 200 mg/3 ml, Phenomenex, Torrance, CA, USA). Chromatographic separation was accomplished on a reversed-phase column (Si-C18, 5 μm, 200 × 0.5 mm i.d.) using a gradient of acetone in an aqueous heptafluorobutyric acid solution. Tandem mass spectrometric detection was performed on a quadrupole-quadrupole linear ion trap instrument (3200 Q Trap, AB Sciex, Foster City, CA, USA) scanning for multiple transitions. Validation included the assessment of selectivity, linearity of the calibration model, accuracy and precision, limit of quantification, recovery and matrix effects, processed sample stability, freeze and thaw stability, and carryover. Within the concentration range 5 to 200 ng/ml, intra- and inter-day precision and accuracies were always better than 15%. A more detailed description can be found elsewhere (Beer et al. 2012).

#### Determination of LH, FSH, SGBH und Estradiol

LH and FSH were measured in serum samples using the IMMULITE 2000 LH and IMMULITE 2000 FSH chemiluminescent immunometric assay kits (Siemens Healthcare Diagnostics, Llanberis, UK), respectively. Analytical sensitivities were 0.05 mIU/mL for LH and 0.1 mIU/mL for FSH. Mean intra-assay coefficients of variation were 3.8% for LH and 3.4% for FSH; mean inter-assay coefficients of variation were 9.1% for LH and 5.4% for FSH. SHBG was measured in serum samples using the IMMULITE 2000 SHBG chemiluminescent immunometric assay (Siemens Healthcare Diagnostics, Llanberis, UK). Analytical sensitivity was 0.02 nmol/L. Mean intra-assay coefficient of variation was 3.1%, inter-assay coefficient of variation was 5.0%. 17β Estradiol was measured in serum samples using a COBAS electro-chemiluminescence Estradiol II immunoassay (Roche, Mannheim, Germany). Limit of quantification was 12 ng/L. For human serum, intra-assay coefficients of variation ranged from 1.7 to 3.3%, inter-assay coefficients of variation ranged from 2.2 to 4.7%. Patients undergoing anastrozole treatment are expected to show estradiol levels below the limit of quantification.

### Statistical analysis

Sample characteristics are presented as frequencies, means, standard deviations and ranges. Variables were scrutinized for their distribution (normality) by use of the Shapiro Wilk test, descriptive parameters (mean, median, skewness) and the histogram. For the non-normally distributed variables SHBG and BMI a logarithmic transformation was applied to achieve an approximately normal distribution. Estradiol was measured as a continuous variable as the essay allows indicating values equal to and above 12 ng/L. For our analysis we grouped the variable dichotomously (≤12 vs. >12 ng/L) as the majority of patients showed values below the limit of quantification (12 ng/L). The overall association of BMI with estrogen and gonadotropin plasma concentrations was analyzed using spearman rank correlation (also for parametric variables to provide consistency across tests). We further investigated the predictive value of BMI for gonadotropin plasma levels under consideration of age using a linear regression analysis. For the prediction of estrogen (grouped variable) levels by BMI we used a logistic regression model. In a second step, anastrozole plasma levels were included in the regression models as independent variable. A *p* value of <0.05 was considered significant.

### Sample size consideration

A sample size of 135 is sufficiently large to detect, under standard conditions regarding type-one error (alpha = 0,05) and statistical power (1-beta =0.8), correlation coefficients above 0.238 or below −0.238. Sample size calculation was performed by use of G-Power 3.1.7.

## Results

### Patient characteristics

Patients (*n* = 135) were aged 65.3 years on average (range 47-85 years) and had been undergoing endocrine treatment for a mean duration 29.5 (SD 17.3) months. Median BMI was 25.5, with a range of 24.2. Please find further details on clinical and sociodemographic patient characteristics in Table 1.

**Table 1:** Clinical and sociodemographic patient characteristics

		*n* = 135
		Frequency (%)
Age	Mean (SD)	65.3a (8.5a)
	Range	47-85
Marital status	Single	8 (5.9%)
	Partnership, marriage	70 (51.9%)
	Divorced, separated	20 (14.8%)
	Widowed	18 (13.3%)
Employment status	Full time	7 (5.2%)
	Part time	9 (6.7%)
	Unemployed	3 (2.2%)
	Homemaker	18 (13.3%)
	Retired	76 (56.3%)
	Other	3 (2.2%)
Diagnosis	in situ	13 (9.6%)
	invasive	121 (89.6%)
Duration of adjuvant endocrine therapy (months)	Mean (SD)	29.5 (17.3)
	Range	2.5-70.8
Primary surgical treatment	Breast conserving surgery	87 (64.9)
	Mastectomy	47 (35.1)
Chemotherapy	yes	27 (20%)
Radiotherapy	yes	98 (72.6%)
BMI	Mean (SD)	25.2 (4.3)
	Median	25.2
	range	19.5-43.7
Estradiol	≤12	119 (88.1%)
	>12	16 (11.9%)
	Median	12 ng/L
	Minimum-Maximum	<12 -69 ng/L
LH	Mean (SD)	26.5 (10.2)
	Median	25.6 U/L
	Minimum-Maximum	0.1-62.10 U/L
	range	62 U/L
FSH	Mean (SD)	83.3 (28.0) U/L
	Median	81.4 U/L
	Minimum-Maximum	26.40-188 U/L
	range	161.6 U/L
SHBG	Mean (SD)	50.3 (20.2) nmol/l
	Median	45.5 nmol/l
	Minimum-Maximum	12.3-116 nmol/l
	range	103.7 nmol/l

### Analysis of anastrozole serum levels

Measurement of anastrozole concentrations in serum showed a median level of 32.8 ng/ml (range = 76.08 ng/ml, Min: 10.42 ng/ml, Max. 86.5 ng/ml). The compound was detected in all patients.

### Association of estrogen and gonadotropin plasma levels with BMI

**Fig. 1:**
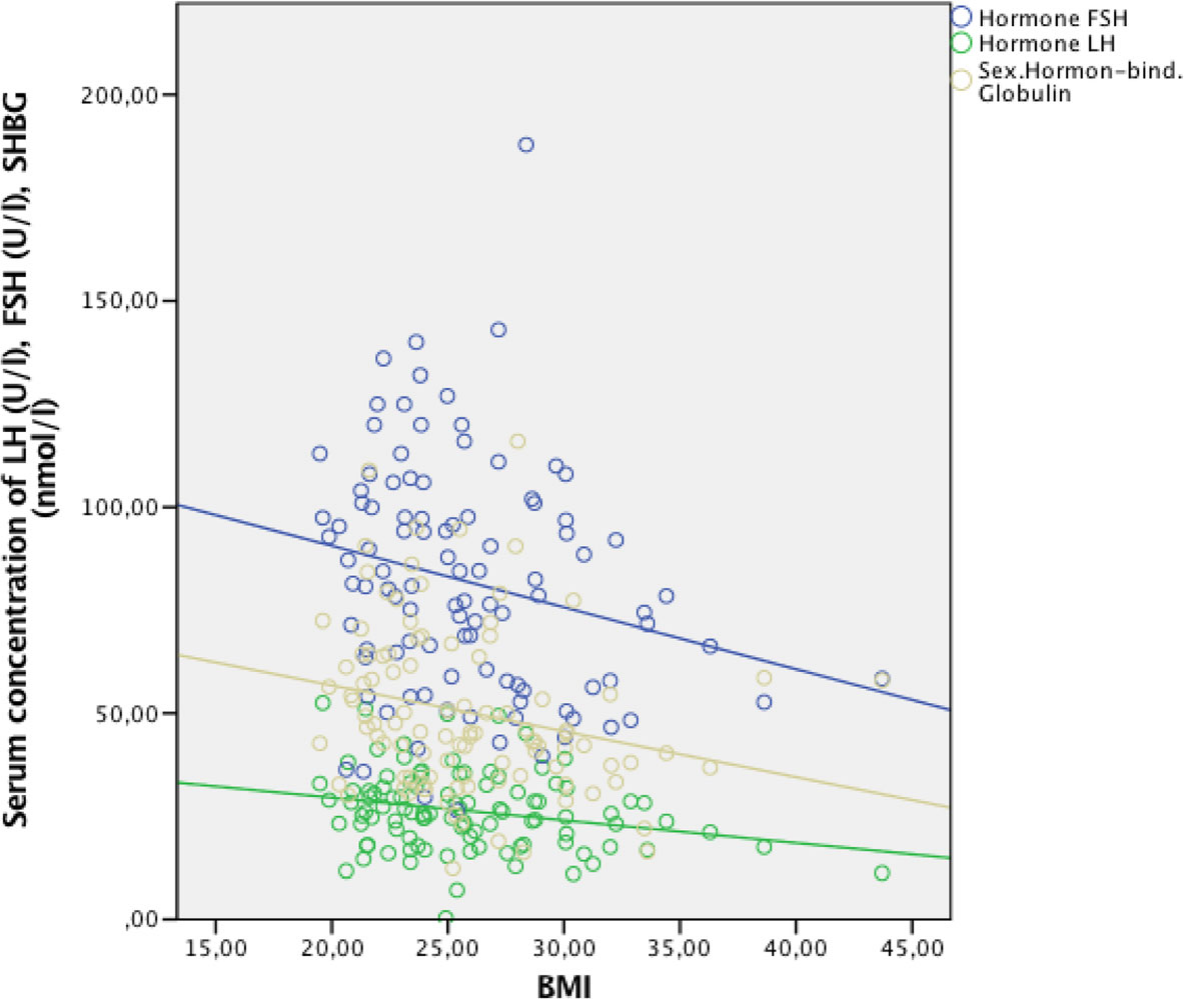
Association of BMI with gonadotropins (LH, FSH) and SHBG (*n* = 135) determined by use of Spearman Rank Correlation

We found a significantly negative correlation of BMI with LH, FSH and SHBG (Fig. 1). BMI and estradiol were not significantly correlated (see Table 2). However, this might be due to limitations in the analysis of estradiol levels.

**Table 2:** Correlation of BMI with estradiol and gonadotropins (*n* = 135)

Hormones	Correlation with BMI (Spearman rank correlation coefficient)	*p*-value
Estradiol	0.004	0.966
LH	- 0.225	0.018
FSH	- 0.260	0.006
SHBG	- 0.312	0.001

LH, FSH and SHBG did not achieve a correlation at a 0.05 significance level with estradiol but a considerable trend towards significance was observed for FSH and SHBG (FSH: *r* = −0.147, *p* = 0.088; SHBG: *r* = −0.165, *p* = 0.063). Again this observation is a result of the limited sensitivity of the estradiol assay.

In a linear regression analysis we investigated the joined effect of BMI and age on LH, FSH and SHBG. BMI significantly predicted levels of LH and SHBG as follows: Per unit increase of BMI, LH decreased by an average of 0.562 units and SHBG by 2.4%, respectively. As expected, a lack of association of BMI and estradiol was confirmed also in the logistic regression analysis. We did not find an impact of age on LH and SHBG. However, after controlling for age in the model for FSH, BMI failed to reach statistical significance but stayed in the model showing a trend that approached significance (*p* = 0.055). In accordance with expectations no predictive value of age in addition to BMI on estradiol concentrations was found. Please find details in Tables 3 and 4.

**Table 3:** Linear Regression model with the independent variables BMI and age

Dependent variable			Regression coefficient		ANOVA	
		Unstandardized beta	Standardized beta	*p*-value	F	*p*-value
LH					7.472	0.007*
	BMI^a^	−0.562	−0.255	0.007*		
	Age			0.838		
FSH					6.862	0.002*
	BMI^a^	−1.2	−0.181	*0.055*		
	Age	−0.837	−0.252	0.008*		
SHBG^a^					6.364	0.013*
	BMI^a^	−0.024	−0.245	0.013*		
	Age			0.132		

^a^Logarithmic transformed

*considered significant indicated by a significance level *p* ≤ 0.05

**Table 4:** Logistic Regression model with the independent variables BMI and age

Dependent variable		Regression coefficient		ANOVA	
		Wald	*p*-value	F	*p*-value
Estradiol				--	--
	BMI^a^	0.18	0.671		
	Age	0.267	0.605		

^a^ Logarithmic transformed

Also the addition of anastrozole plasma concentration as an independent variable did not contribute to the regression models.

Finally, analysis revealed a trend towards significance for the association of estradiol with FSH (*r* = −0.147, *p* = 0.088) and SHBG (*r* = 0.-165, *p* = 0.063) but not for LH (*r* = 0.00, *p* = 0.990).

## Discussion

Several studies have identified BMI as a crucial factor for BC genesis, cancer subtype, treatment response and finally outcome [6, 14–18]. This observation probably originates from increased estrogen plasma concentrations related to overweight [7]. Regarding endocrine treatment with AIs recent evidence suggests that also during the intake of standardly dosed AIs these increased estrogen levels persist and, thus, contribute to the reduction of treatment efficacy [3, 5, 8]. However, these results are currently controversially discussed [19]. Studies available are limited by standard measurement methods for estradiol which lack functional sensitivity [9]. Additionally, these methods are usually cost intensive. There is a lack of data on other biological effects of anastrozole beside estrogen suppression [10].

The present study was subjected to getting a more comprehensive picture of the impact of BMI on the hormonal state in postmenopausal patients receiving anastrozole in routine clinical care. We assumed that gonadotropins (LH, FSH, SHBG) might act as surrogate marker for estrogenic activity and also considered anastrozole serum concentrations as a mediating factor. We also hypothesized that estradiol levels are less important than “estrogenic activity”, which is defined as any stimulation of the estrogen receptor by various molecules including precursors and metabolites of estradiol. There is also accumulating evidence that various agents and drugs may also exhibit biological activity on the estrogen receptor. This means that several, yet unknown compounds besides steroid hormones might have activity on the estrogen receptor in patients under aromatase inhibitor treatment (AT).

In fact, we found serum gonadotropin levels (LH, FSH and SHBG) [9] to be significantly lower in obese women compared to normally weight women under AT. This result persisted independently of age. Since gonadotropins are expected to increase in association with decrease of estrogen it can be assumed that BMI impacts on estrogenic activity under AT. So far, obesity has been well-known to be related to higher estrogen serum concentrations only in women without a BC diagnosis/treatment [10, 18], yet under endocrine treatment an approximation to homogenous estrogen and gonadotropin levels of serum concentrations had been expected regardless of BMI. According to our results postmenopausal, obese women, still have a differing endocrine situation compared to normally weight women despite receiving anastrozole treatment (AT). Our results are supported by a recent study of Pfeiler and colleagues [20] who prospectively investigated levels of estradiol suppression related to BMI as well as FSH serum concentrations in adjuvant-treated postmenopausal patients with BC. The authors also found significantly lower FSH levels in obese women compared to normally weight women. Still, a very recent study determining estradiol over a period of 24 months showed contradictory results [21]. The initial, well-known difference of estradiol serum concentrations between overweight and normally weight women did not persist to the 12- and 24-month follow-up assessment under endocrine treatment. However, overweight women had increased estradiol levels at all time-points. Results thereafter might be influenced by a quite small overall sample size of 70. Information on gonadotropin levels might have had additional explanatory benefit.

Another notable finding herein is that we were able to demonstrate that levels of anastrozole serum concentrations do not have a mediating effect on estrogenic activity. This is particularly of importance since anastrozole serum concentrations proved to be related to BMI in this patient sample. Detailed results in this regard have been presented [6]. This again strengthens the significant role of BMI with regard to the patient’s hormonal situation under AT.

We did not find a significant association of BMI and estradiol measured by a routine laboratory assay. This result was somehow expected and can be explained by two major reasons: First, it has been previously reported that serum estradiol levels do not correlate with BMI [3, 7, 17] and estradiol measurement within breast tissue might better reflect aromatase activity. Second, we lacked a hypersensitive assay for estradiol measurement and derived the hormonal data from the routine laboratory examination report based on an assay with low sensitivity.

However, the measurement of estradiol has been previously challenging [9]. Mass spectrometry methods seem to be currently the only estradiol assays showing satisfactory sensitivity; but this method is labor- and cost- intensive and difficult to integrate into clinical routine. Finally, also Pfeiler and colleagues were not able to illustrate a significant association of BMI and levels of estradiol [20].

## Conclusion

In view of our results, the previously observed impact of BMI on the hormonal situation in women under AT seems to be reflected also by gonadotropin concentrations; as we had a adequately powered sample and were able to exclude confounding factors such as anastrozole serum concentrations our findings might further support the notion [3, 20] that estrogens are insufficiently suppressed in obese women.

At this point, our study findings are a further indicator for the relevance of BMI in regard of anastrozole metabolism and in conjunction treatment efficacy. Though not answered yet, the question whether increased estrogen levels in obese women undergoing AT are responsible for a reduced treatment efficacy has been brought forward. Other potential explanatory factors such as insulin, adipocytokines such as leptin, and adiponectin, as well as inflammatory markers such as C-reactive protein and interleukins will have to be additionally investigated. We suggest the observation of gonadotropin plasma concentration to be a cheap and useful surrogate parameter in clinical routine for the monitoring of patients’ treatment response. However, still more studies are necessary to implement this observation to clinical practice.

## Abbreviations

AI: Aromatase inhibitor
AT: Anastrozole treatment
BMI: Body mass index
FSH: Follicle-stimulating hormone
LH: Luteinizing hormone
SHBG: Sex hormone-binding globulin
